# Cardiorespiratory function, resting metabolic rate and heart rate variability in coal miners exposed to hypobaric hypoxia in highland workplace

**DOI:** 10.7717/peerj.13899

**Published:** 2022-08-30

**Authors:** Sanjun Yang, Chunhu Tian, Fan Yang, Qi Chen, Ruiyuan Geng, Chunyan Liu, Xinrong Wu, Wing-Kai Lam

**Affiliations:** 1 Department of Physical Education, China University of Mining and Technology-Beijing, Beijing, China; 2 Sports Science Research Center, Li Ning Center, Beijing, China; 3 The University of International Business and Economics, Beijing, China; 4 The Third Affiliated Hospital of Beijing University of Chinese Medicine, Beijing, China; 5 School of Emergency Management and Safety Engineering, China University of Mining and Technology-Beijing, Beijing, China; 6 Sports Information and External Affairs Centre, Hong Kong Sports Institute, Sha Tin, Hong Kong, China

**Keywords:** Miners, Highland, Resting state, Cardiorespiratory function, Resting metabolic rate, Heart rate variability

## Abstract

**Background:**

Owing to intermittent/acute exposure to hypobaric hypoxia, highland miners may often suffer, the physiological characteristics between highland and lowland miners, however, are rarely reported. The objective of this study was to compare the physiological characteristics of coal miners working at disparate altitudes.

**Methods:**

Twenty-three male coal mining workers acclimating to high altitude for 30 ± 6 days in Tibet (highland group; approx. 4500 m above sea level; 628.39 millibar), and 22 male coal mining workers in Hebei (lowland group; less than 100 m above sea level; 1021.82 millibar) were recruited. Tests were conducted to compare ventilatory parameters, circulation parameters, resting metabolic rate (RMR), and heart rate variability (HRV) indices between the two groups in resting state.

**Results:**

Ventilation volume per minute (VE) of the highland group was markedly raised compared to that of the lowland group (11.70 ± 1.57 vs. 8.94 ± 1.97 L/min, *p* = 0.000). In the meanwhile, O2 intake per heart beat (VO2/HR) was strikingly decreased (3.54 ± 0.54 vs. 4.36 ± 0.69 ml/beat, *p* = 0.000). Resting metabolic rate relevant to body surface area (RMR/BSA) was found no significant difference between the two groups. Evident reduction in standard deviation of NN intervals (SDNN) and remarkable increase in ratio of low- and high- frequency bands (LF/HF) were manifest in highland miners compared to that of lowland ones (110.82 ± 33.34 vs. 141.44 ± 40.38, *p* = 0.008 and 858.86 ± 699.24 vs. 371.33 ± 171.46, *p* = 0.003; respectively).

**Conclusions:**

These results implicate that long-term intermittent exposure to high altitude can lead miners to an intensified respiration, a compromised circulation and a profound sympathetic-parasympathetic imbalance, whereas the RMR in highland miners does not distinctly decline.

## Introduction

The number of mining workers exploiting kinds of mineral resources at altitudes has been increasing. Highland miners are commonly subjected to periodically alternating workplaces between altitude and lowland, characterized of being exposed to hypobaric hypoxia intermittently and acutely.

The Tibet Plateau has an average altitude of 4,500 m above sea level. The Qinghai-Tibet Plateau, is the highest altitude in the world, known as the “roof of the world”. Environment at high altitudes is characterized of thin air, low atmospheric pressure, strong ultraviolet light, dry and windy climate (Gaur et al., 2021).

Individual who sustains an acute exposure to the hypobaric hypoxic environment can elicit a systemic and extensive stress responses (Zouboules et al., 2018). The respiratory status is affected by neurohumoral regulation and translates rapidly into hyperventilation (Tansey, 2008; Siques et al., 2020), which emerges within few minutes of exposure to hypoxia and maintains throughout the exposure (Imray et al., 2010). Hyperventilation provokes an imbalance of alkali and acid reserves, the body subsequently developing into respiratory alkalosis (Parati et al., 2018; Zouboules et al., 2018). In terms of coal miners, even if pneumoconiosis is not detected prior to resign/retirement, and no more subsequent exposure to dust after leaving, lung tissue still has the possibility of progression to fibrosis (Almberg et al., 2020).

Highland exposure may also cause the reduction of arterial oxygen partial pressure and the decreased blood oxygen saturation, which leads to insufficient systemic oxygen supply (Imray et al., 2010; San et al., 2013) . The stimulation of hypoxia triggers major changes in cardiovascular function (Naeije, 2010). Firstly, cardiac output is augmented with tachycardia, no change in stroke volume, but blood pressure may temporarily slightly increase, and after a few days of acclimatization, cardiac output then reverts to baseline, heart rate remaining increased, stroke volume hence decreased (Naeije, 2010). Subsequently, blood properties are altered in response to hypoxia: one is to undergo in succession dehydration-induced plasma volume reduction, erythropoietin (EPO) secretion increase, polycythemia and eventually an elevated hemoglobin concentration, hence blood viscosity rising (Naeije, 2010; Bogaard et al., 2002). Aerobic performance basically depends on the matching conditions of the heart, lung, blood, blood vessels and tissue cells, which can mirror the overall physiological processes of oxygen diffusion, transfer and ultimate utilization in the tissue mitochondria (Storz & Cheviron, 2021). Heart rate (HR) and O_2_ intake per heart beat (O_2_/HR, namely O_2_ pulse) are non-invasive, sensitive and effective indicators as to reflect cardiac status and the general situation of O_2_ consumption.

Basal metabolic rate (BMR) is the minimum rate of energy expenditure or the compulsory energy cost to keep an individual himself alive. In adult humans, a large proportion (i.e., ∼60) of the energy budget is allocated to the BMR to maintain body functions such as blood flow, breathing and tissue activities (Careau, 2017). As is extensively acknowledged, illness and disease, to a great degree, may magnify BMR (Henry, 2000). Similarly, the human body exposed to hypoxia needs to bear more physiological stresses and struggles to maintain homeostasis, consequently affecting BMR. The increased BMR is proposed to be one of the causes of the altitude body weight loss (San et al., 2013; Westerterp & Kayser, 2006). In this research, we employ resting metabolic rate (RMR) as an approximately expression of BMR for the sake of accuracy.

Hear rate variability (HRV) refers to the temporal changes in the beat-to-beat intervals in the heart, which is subject to the autonomic nervous system (ANS) and reflects the competition for the predominance between sympathetic and parasympathetic nerves (Boos et al., 2018). HRV is at present widely applied to the evaluation of psychological stress, physical fitness and prevention of over training (Sassi et al., 2015). In addition, a previous study has revealed the relationship between fatigue and HRV parameters (Chen et al., 2020). Still, suicide idea is associated with vagal control (Adolph et al., 2018), and also, decreased HRV is related to increased immunometabolic risk (Hu et al., 2018). Since altitude hypoxia imposes systemic potent impacts on individuals, plenty of studies have probed the malregulations of ANS though HRV following exposure to high altitudes (Hamm et al., 2018). Generally, HRV consists of three categories of indices: time-domain, frequency-domain and nonlinear indices. Time-domain parameters are derived from the linear analysis of successive RR interval sequences, whilst frequency-domain parameters stem from the distribution of power frequency (Chen et al., 2020). Nonlinear analysis based on chaos theory is proposed to determine cardiac dynamic state. In this study, standard deviation of NN intervals (SDNN) and root mean square of successive RR interval differences (RMSSD) are employed regarding parameters of time-domain. Additionally, frequency-domain parameters adopted are as follows: very low frequency (VLF), low frequency (LF), high frequency (HF) and ratio of low- to high- frequency (LF/HF). HF is said to implicate parasympathetic activity and LF, deemed as a marker of both sympathetic and parasympathetic activities, refers to predominantly sympathetic activity (Insalaco et al., 2016).

At present, it is relatively sparse that investigations, aimed at the specific population of miners, into physiological responses to highland familiarization involve cardiorespiratory function, RMR and HRV collectively. The purpose of this project is to compare the physiological parameters of highland and lowland miners using portable cardiopulmonary function meter and 72-h single-lead ECG recording module. We intend to clarify the mechanism of altitude impairments on highland miners. The results from this study would provide some quantitative clues for clinical practice.

## Materials & Methods

### Materials

A total of 45 male miners was recruited in the study: 23 in Tibet (highland group; approx. 4500 m above sea level; 628.39 millibar), and 22 in Tangshan, Hebei Province (lowland group; less than 100 m above sea level; 1021.82 millibar), respectively. Their baseline demographic characteristics are summarized in Table 1. Both groups of participants were of the same ethnicity: Chinese Han, born and raised at lowlands (≤ 600 m) and sharing similar working years on the same position (4 ± 1 years). The routine work of both groups was to manipulate mining machines underground, in which they engaged in moderate labor. Specifically, the high and low groups worked approximately 500 m and 750 m deep below the surface. All of them underwent medical examinations twice a year in local hospitals according to the regulations of the company. Before they were enrolled in our study, they met company’s health requirements. Additionally, those who had experienced the following conditions by self- report were to be excluded in our study: cardiopulmonary disorders, hepatobiliary diseases, blood diseases, diabetes, hypertension, neurological disorders, nephropathy, thyroid disorders or metabolic syndrome. No participants administered medications for at least one week before the study, and they were also asked to refrain from alcohol, tea or caffeinated drinks at least 3 days prior to the study. All the participants complied with a circadian rhythm that they went to bed at 23:00. They were informed of the process of the test and the physiological indicators to be collected. Verbal informed consent was acquired from all the participants. The study was approved by the ethics committee of China University of Mining & Technology-Beijing, Beijing, China, which was performed in accordance with the guidelines of the Declaration of Helsinki and Belmont Report.

**Table 1:** Demographic characteristics of the participants included in the study.

	Lowland	Highland	*P*
Number	23	22	
Age(yr)	36.91 ± 5.49	35.41 ± 6.74	0.415
Height(cm)	171.39 ± 6.17	173.36 ± 5.26	0.256
Weight(kg)	70.89 ± 11.61	79.93 ± 10.97	0.010
BMI(kg/m^2^)	24.04 ± 3.25	26.57 ± 3.24	0.012

**Notes.**

Data format is mean ± SD (standard deviation).

BMI body mass index

### Determination of cardiorespiratory parameters in resting state and RMR

Neither exhausting exercise within 24 h, nor food consumption within 12 h prior to the RMR measurement was allowed (Slinde et al., 2008; Bowes, Burdon & Taylor, 2021), but normal fluid intakes was permitted during the fast (Bowes, Burdon & Taylor, 2021). Participants fulfilled a 10-minute supine rest on the bed firstly, and the recording afterwards initiated. During the recording, they were instructed to remain the supine position on the bed, keeping stationary, silent and awake in a quiet and low illumination room (∼22 °C; ∼50% relative humidity) (Bowes, Burdon & Taylor, 2021). Gas metabolism analyzer (MetaMax3B-R2; Cortex, Leipzig, Germany) was employed to determine tidal volume (VT), breathing frequency (BF), ventilation per minute (VE), oxygen intake (VO_2_) and RMR simultaneously in a complete quiescent state of the participants. The device was able to link heart rate belt (H7, Polar, Finland) via Bluetooth so as to collect HR. The variables mentioned above were averaged from 5-minute episode out of a total 25-minute recorded duration for each participant through the gas metabolism analyzer exclusive software. Gas metabolism analyzer was calibrated thoroughly prior to measurements. In order to obtain the derivative parameter RMR/BSA, body surface area was calculated with the Mosteller body surface area equation (Mosteller, 1987): BSA (m^2^) = sqrt {[height (cm) * weight (kg)] / 3600}.

### Measurement of HRV

Data acquisition was performed via beat-to-beat heart rate monitoring instrument (Bodyguard II; Firstbeat, Jyväskylä, Western Finland, Finland). After Preparing the skin with an alcohol cotton swab, the electrodes (diameter 50 mm; impedance less than 3k Ω) were pasted at the positions following the apparatus’ manual (On the right mid-clavicular line, slightly below the clavicle; on the left anterior axillary line, at the horizontal level of the left mid-clavicular line in the fifth intercostal space; respectively). The discharge course of heart was recorded and stored into the receiving module put on the chest through electrodes and leads. This equipment could continuously record data for 72 h. Each group of the participants collected 72 h of continuous data, excluding the data with too many artifacts. Time course segments on each day selected for different phases of HRV calculations (i.e., diurnal phase and nocturnal phase) were 6:00–12:00 and 0:00–6:00. Exclusive analysis software facilitated the HRV calculations.

### Statistical analysis

SPSS version 26 mathematical statistics software was employed. The data were expressed as Mean ± SD (mean ± standard deviation). The Levene’s test was applied for equality of variances. Except that BF and LF/HF did not satisfy the equality of variances, the other reported variables did. The differences in the mean between the two groups were assessed by 2-tailed independent sample T test. The degrees of freedom for BF and LF/HF were 31.38 and 24.75, respectively, and the degree of freedom for other reported variables was 43. The significant level was set at *p* < 0.05.

## Results

**Figure 1:**
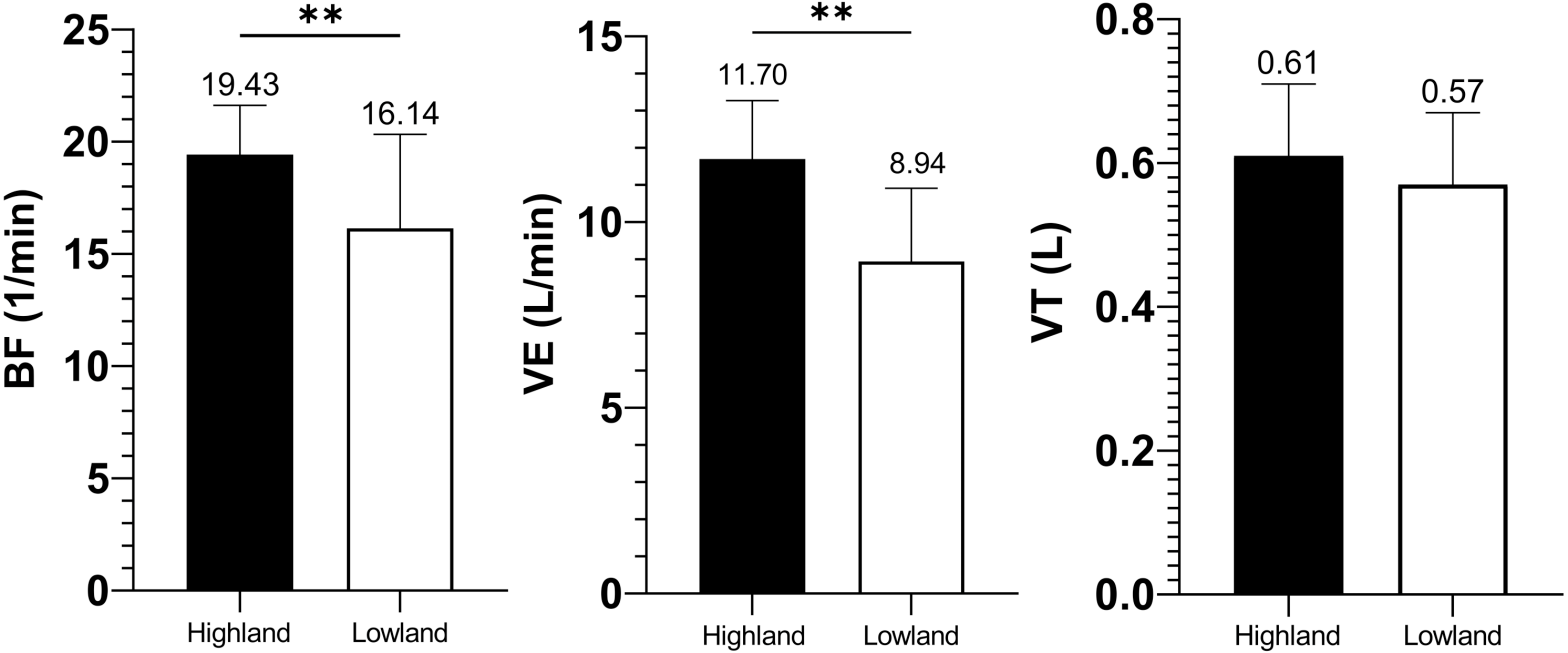
Ventilation variable comparisons of BF, VE and VT.

The age and height showed no significant difference between highland and lowland miners, but significant differences existed in the body weight and BMI (shown in Table 1). Both BF and VE of the highland group were significantly higher than those of the lowland group (19.43 ± 2.19 vs. 16.14 ± 4.19 1/min, *p* = 0.002 and 11.70 ± 1.57 vs. 8.94 ± 1.97 L/min, *p* = 0.000; respectively), yet tidal volume (VT) of highland miners showed no statistically difference in comparison with that of lowland ones (0.61 ± 0.10 vs. 0.57 ± 0.10 L, *p* = 0.182), as presented in Fig. 1.

**Figure 2:**
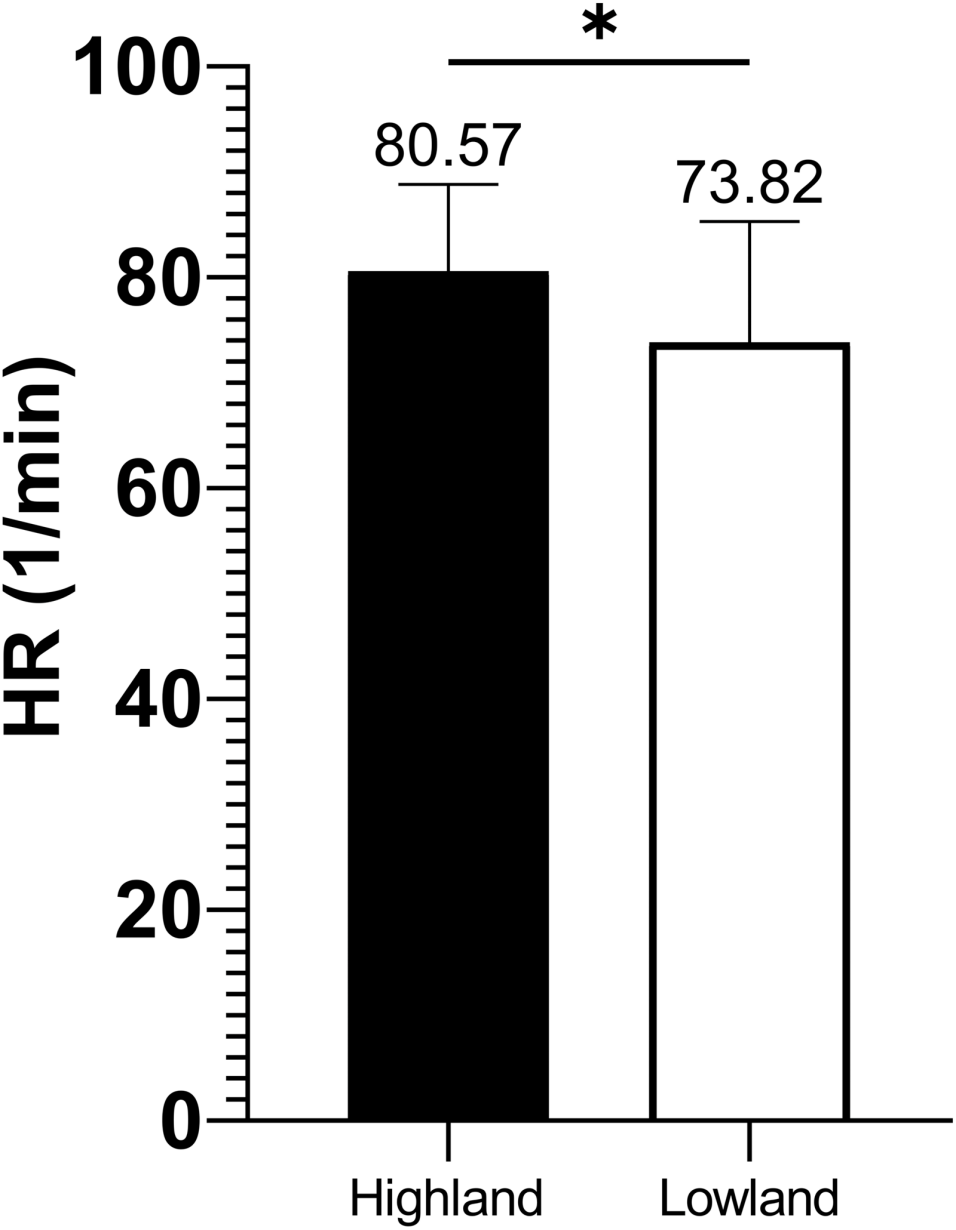
HR comparison between highland and lowland groups.

**Figure 3:**
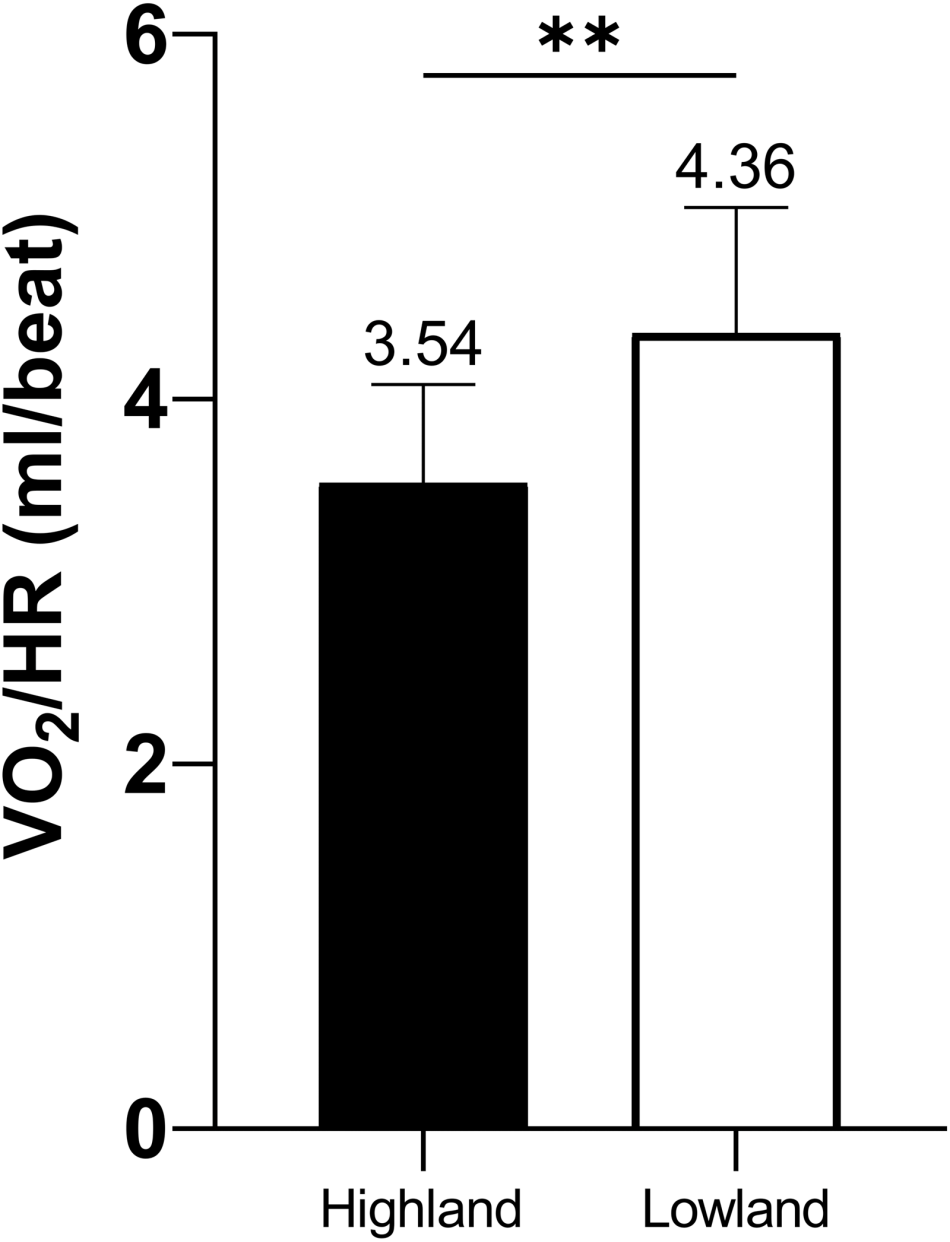
VO_2_/HR comparison between highland and lowland groups.

HR was significantly higher in the highland group compared to that of the lowland group (80.57 ± 8.25 vs. 73.82 ± 11.48 beat/min, *p* = 0.028), as shown in Fig. 2, whilst VO_2_/HR was strikingly decreased compared to that of the lowland group (3.54 ± 0.54 vs. 4.36 ± 0.69 ml/beat, *p* = 0.000), as shown in Fig. 3.

**Figure 4:**
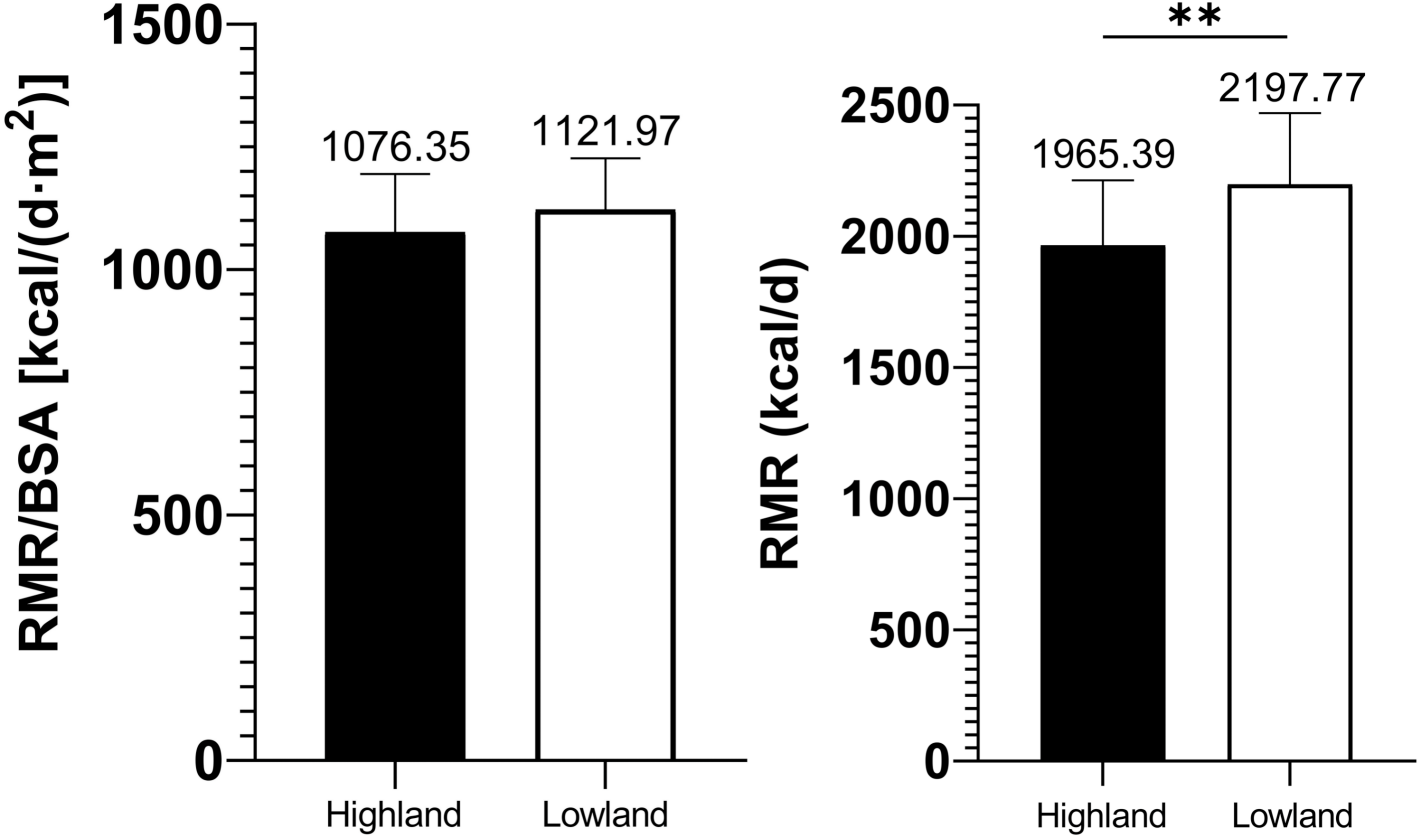
Comparisons of relative and absolute resting metabolic rate amid highland and lowland groups.

RMR/BSA in highland miners presented no significant difference in contrast to that of lowland ones (1076.35 ± 118.27 vs. 1121.97 ± 104.63 kcal/(d m^2^), *p* = 0.178), albeit the prominently less amount of RMR in the highland group (1965.39 ± 248.10 vs. 2197.77 ± 271.47 kcal/d, *p* = 0.004), which were summarized below in Fig. 4.

RMSSD (asleep state) and SDNN in highland miners were found considerably lower than that of lowland ones (29.25 ± 12.22 vs. 40.64 ± 21.28, *p* = 0.032 and 110.82 ± 33.34 vs. 141.44 ± 40.38, *p* = 0.008; respectively). Furthermore, LF/HF in highland miners manifested a significant increase (858.86 ± 699.24 vs. 371.33 ± 171.46, *p* = 0.003). Nevertheless, HF of the highland group was far less in contrast to that of the lowland group, but the difference had not yet reached the statistical significance (show in Table 2).

## Discussion

Upon being exposed to altitude hypoxia (highland, 4500 m above sea level), the initial and immediate consequence is the reduced arterial oxygen partial pressure resulting in arterial carbon dioxide and H^+^ concentration elevated in the body (Frisancho et al., 1999). Respiration outcomes in our study indicated altitude hypoxia enhanced the ventilation drive, thus hyperventilation. This coincides with the mechanism by previous studies which propose that peripheral chemoreceptors, mainly carotid body, and central chemoreceptors are excited, and the impulse of the peripheral chemoreceptors travels along the sinus nerves and vagal nerves to the respiratory center, regulated by the respiratory center, thus increased respiratory frequency and reinforced ventilation (San et al., 2013; Smith et al., 2017). It was observed that VE and BF were significantly elevated in highland miners compared with those of lowland ones, and this is consistent with previous studies. However, no significant difference was found in VT between the two groups, which does not coincide with previous reports. This disparity might be explained by the distinct body weights of the two groups. When normalized by body weight, VT per kg in highland miners was significantly increased compared to that of lowland ones (8.71 ± 1.58 vs. 7.14 ± 1.08 ml/kg, *p* = 0.000). Thus, highland miners might have a tendency towards higher VT in resting state, but further investigation is needed before a viable conclusion can be drawn. Associated the particular environment of mining with hyperventilation, it can be inferred that highland miners are potentially more vulnerable to pulmonary diseases. It was reported that the development and progression of pneumoconiosis were accelerated at altitudes (Voznesenskii˘, 1996), and this finding has proved our inference.

**Table 2:** Comparison of heart rate variability between the two groups.

	Highland	Lowland	*P*
RMSSD(asleep state)	29.25 ± 12.22	40.64 ± 21.28	0.032
RMSSD(awake state)	18.93 ± 6.92	22.55 ± 13.61	0.264
SDNN	110.82 ± 33.34	141.44 ± 40.38	0.008
LF/HF	858.86 ± 699.24	371.33 ± 171.46	0.003
LF	1144.14 ± 555.85	1100.46 ± 576.53	0.797
HF	568.25 ± 461.03	965.23 ± 897.09	0.067
VLF	165.88 ± 82.45	135.45 ± 68.72	0.187

**Notes.**

Abbreviations: Root mean square of successive RR interval differences (RMSSD); RMSSD derived from daytime and nighttime, RMSSD (awake state) and RMSSD (asleep state), respectively; standard deviation of NN intervals (SDNN); ratio of low- and high- frequency bands (LF/HF); low frequency (LF); high frequency band (HF); very low frequency band (VLF). Values are expressed as means ± SD (standard deviation).

As is well known, systemic stress response increases sympathetic tone (Farias et al., 2013), respiratory frequency (Tansey, 2008), heart rate and shortens the cardiac cycle (Tansey, 2008; Bogaard et al., 2002). The HR of miners who have been at altitude for about one month is still about 8.5% higher than that of lowland ones, suggesting that hypoxia-induced sympathetic stress is predominant and persists (Vearrier & Greenberg, 2011). Our observation of high HR in highland miners is in agreement with previous finding that either life-long altitude natives or lowlanders recently acclimatized to altitude manifest a higher HR associated with sympathetic nervous system activation (Dedobbeleer et al., 2015). Furthermore, accompanied by the acclimatization of plateau, the blood RBC (red blood cell) number and hemoglobin concentration increase, and the hematocrit becomes large (Naeije, 2010; Shahid et al., 2019). Although these changes in blood physicochemical properties improved their oxygen carrying capacity and compensated the profoundly decreased arterial partial pressure of oxygen (PaO_2_) to some extent (Farias et al., 2013), these, however, could not completely offset the O_2_ deficit resulted from decreased PaO_2_ and stroke volume. Hence VO_2_/HR in highland miners was markedly reduced compared to that of lowland ones as was expected.

Basal metabolic rate (commonly depicted roughly through resting metabolic rate, RMR) reflects the energy needed by the body to maintain the basic blood circulation, breathing, body temperature, and internal environmental homeostasis (Burton et al., 2011). In our study, RMR was significantly lower in highland group than that in lowland ones, whereas RMR/BSA demonstrated no significant difference between the two groups. This may be ascribed to the fact that the height and the weight of the highland group are significantly lower than that of the lowland group, and the real resting metabolic status is thus masked. RMR/BSA, therefore, can be more likely to reflect the resting metabolic status in both groups. RMR/BSA in our study suggested that the highland miners had turned into a state of stress response, resulted from sympathetic excitement, accompanied by higher catecholamine and thyroid hormone levels (Farias et al., 2013; Burton et al., 2011), which could positively promote the body’s metabolic rate. Despite the ambient hypoxia, the body’s vigorously adapting to the stressful conditions did not decrease RMR/BSA in the highland group. Farias et al. stated that BMR increased during the first days of exposure to high altitudes, apparently depending on the attained altitude and subsequently decreased as time prolonged, without achieving sea level values (Farias et al., 2013). This is largely consistent with our outcome.

The hypobaric hypoxic stress simultaneously activates both hypothalamic-pituitary-adrenal (HPA) and sympathetic-adrenal medulla (SAM) (Mohammadi et al., 2019), of which sympathetic and parasympathetic nervous systems primarily account for HRV. Sympathetic system consists of sympathetic nerves and adrenal gland: sympathetic neurons synthesize and release noradrenaline as their main neurotransmitter to synaptic clefts, subsequently exerting the effects on postsynaptic membranes, while adrenaline secreted from adrenal gland serves as a hormone, arriving at its target cells and organs via the circulation (Hein, 2015; Seravalle et al., 2018). On the other hand, parasympathetic nerves release acetylcholine as their major neurotransmitter that innervates nicotinic receptors of postganglionic neurons (Hein, 2015; Seravalle et al., 2018; Deepak, 2011). In the heart, sympathetic activation increases HR, conduction velocity, contraction speed, and relaxation, consequently reducing the total HRV, whereas parasympathetic activation decreases HR and atrioventricular conduction, thus causing a rise to HRV (Hein, 2015). The evident elevation of biochemical factors such as cholesterol, triglyceride, high density lipoprotein, low density lipoprotein, homocysteine and catecholamines is considered to be deeply involved in ANS responses to altitude, consequently embodied largely in HRV (Dhar et al., 2018). As a kind of special environments, high altitude imposes much stress on ANS, leading to an elevated level of catecholamines, thus a reduction in HRV. It was revealed that highland natives could regain the resting state more quickly than lowland natives during post exercise recovery through HRV measures, implying highland native’s parasympathetic nerves being reactivated to a greater extent (Bhattarai et al., 2018). Accordingly, in our study, the prominent reduction in SDNN is consistent with previous investigation in which acclimatized lowlanders exhibited a lower SDNN compared to sea level residents and altitude natives (Dhar et al., 2018), suggesting an attenuated vagal together with an intensified sympathetic modulation of sinus node (Lombardi, 2002). Decreased HRV is associated with stress and disease (Wheat & Larkin, 2010). Furthermore, a reduction in HRV indices, particularly of SDNN and triangular index is a convincing predictor of increased morbidity of patients with cardiovascular diseases (Lombardi, 2002; Schmidt, Müller-Werdan & Werdan, 2007). The significant elevation of LF/HF observed in present study is in line with previous findings, which can be interpreted as sympathetic activation being prevailing over parasympathetic activation (Bhattarai et al., 2018; Dedobbeleer et al., 2015; Lombardi, 2002; Lima, Osório & Gamboa, 2020). Moreover, an evident tendency towards a lower HF in the highland group in this study is to an extent a reflective of vagal tone, for vagal activity is influenced by two distinct autonomic pathways of originating in the dorsal motor nucleus and the nucleus ambiguous (Wheat & Larkin, 2010). It has been appreciated that a good HRV score is supposed to be above 0.04 Hz for LF and below 0.26 Hz for HF (Mahmad Khairai, Abdul Wahab & Sutarto, 2022), and besides, LF component of HRV strongly correlates with the plasma levels of norepinephrine (Deepak, 2011). The highland miners in our study assumed an obviously diminished RMSSD (asleep state) compared to the lowland ones, whereas RMSSD (awake state) had not yet reached a significant difference between the two groups. Now that RMSSD is considered to be a surrogate of parasympathetic activation (Deepak, 2011), the markedly reduced RMSSD in highland miners in our study is thereby potentially attributed to the persistence of enhanced sympathetic nervous excitability, and concomitant presence of the consistent inhibition of parasympathetic tone. Therefore, the inter-group RMSSD difference in our study was further pronounced at night.

Several shortcomings of this study need to be acknowledged:

(1) Relatively small sample size. We had to compromise the sample size due to the difficulty in recruiting participants and the restraint of logistics in the context of such a high altitude. It merits greater input of human and material resources so as to enlarge the sample size in the further study.
(2) Significant body weight and BMI differences between the two groups. These directly led to a prominent inter-group difference in absolute RMR, which in our study was corrected via normalized variable RMR/BSA that made the results comparable.
(3) We failed to execute the blood gas analysis of our great interest in this study.

## Conclusions

(I) Prolonged intermittent exposure to high altitude can lead the highland miners to alterations of ventilation, cardiovascular system and HRV.

(II) Chronic intermittent altitude exposure, however, does not sufficiently alter RMR/BSA in present study, at least no noteworthy reduction being observed.

(III) Chronic intermittent altitude exposure renders miners enhanced sympathetic and weakened parasympathetic nervous tones.

(IV) The exacerbated sympathovagal imbalance may be more likely to predispose high altitude miners to cardiovascular diseases and psychological disorders.

(V) What’s more, hyperventilation respiratory pattern probably involves in the high incidence of pulmonary diseases among altitude miners.

Further healthcare and occupational protection are worthy of taking into consideration.

##  Supplemental Information

10.7717/peerj.13899/supp-1 Data S1 Raw data of physiological parameters from the tests of both groups of participants

## Additional Information and Declarations

### Data Availability

The following information was supplied regarding data availability: Raw data are available in the Supplemental Files.
